# Supplementary material for: Exploring barriers and facilitators in implementation fidelity of malaria screening intervention at Nepal-India border point-of-entry health desks-A mixed method study
Source: PLoS One. 2025 May 7;20(5):e0323116. doi: 10.1371/journal.pone.0323116 (PMC12057938; doi:10.1371/journal.pone.0323116)
Supplement: S1 File — xxx. (ZIP) [file pone.0323116.s001.zip › Data collection tools and guideline.docx]

# Observation Checklist

| **S.N** | **Particulars** | |
| --- | --- | --- |
| 1 | Name of the point of entry Health Desk: |  |
| 2 | Opening and closing hours: |  |
| 3 | Availability of the SOP/guideline: (Yes/No) |  |
| 4 | Total staffs available in one shift: |  |
| 5 | Time shift per staffs: |  |
| 6 | Type of screening: (Entry/exit) |  |
| 7 | Physical Attributes of the health desk (Concrete/ Temporary) |  |
| 8 | Frequency (Routine/ad hoc) |  |

|  |  | **Y** | **N** |  | **Y** | **N** |
| --- | --- | --- | --- | --- | --- | --- |
| **Before screening** | Availability of police officer | □ | □ | Availability of Community Health Workers | □ | □ |
|  | Availability of IEC materials | □ | □ |  |  |  |
| **Methods** | Questionnaire (residence, occupation and travel history) | □ | □ | Body temperature check | □ | □ |
| **Counseling** | Clear communication | □ | □ | Culturally sensitive and free from discrimination | □ | □ |
|  | Language support | □ | □ |  |  |  |
| **Safety:** | Sharps container | □ | □ | Gloves | □ | □ |
|  | Biohazard waste bin/bag | □ | □ | Hand hygiene | □ | □ |
|  | Exposure prophylaxis | □ | □ | N95 Masks/surgical | □ | □ |
| **Remarks:** |  |  |  |  |  |  |

**PERFORMANCE OF PROCEDURES**

|  |  | **Y** | **N** |  | **Y** | **N** |
| --- | --- | --- | --- | --- | --- | --- |
| **Preparation** | Label initials onto cassette | □ | □ | Check lot number | □ | □ |
|  | Correct use of timer | □ | □ | Check expiration date | □ | □ |
|  |  | Y | **N** |  | Y | **N** |
| **Finger prick** | Sterile lancet | □ | □ | Correct position of prick | □ | □ |
|  | Correct massage | □ | □ | Dispose lancet in sharps | □ | □ |
|  | Correct disinfection | □ | □ | Correct wiping of first drop | □ | □ |
|  |  |  |  | Sufficient volume of blood using 1 prick (4 tests) | □ | □ |
| **Remarks:** |  |  |  |  |  |  |

|  |  | | **Y** | **N** |  | **Y** | **N** |
| --- | --- | --- | --- | --- | --- | --- | --- |
| **Malaria Testing:** | Malaria pipette | | □ | □ | Deposit all blood onto sample well *touching* **sample pad** | □ | □ |
|  | Malaria buffer | | □ | □ | Dispose pipette in sharps | □ | □ |
|  | Malaria cassette | | □ | □ | Correct number of buffer drops into **buffer well** | □ | □ |
|  | Correct pipetting | | □ | □ | Starting timer immediately after buffer | □ | □ |
|  | Sufficient volume drawn to line (5µl) | | □ | □ | Waiting full time to read -ve result | □ | □ |
|  | Correctly interpret result and record | | □ | □ | Discard gloves and cassette in non-sharp waste container | □ | □ |
| **Remarks:** |  | |  |  |  |  |  |
|  | |  | Y | **N** | Quantity | | |
| **Supply** | | RDT per day |  |  |  | | |
|  | | Antimalarial for P. falciparum | □ | **□** |  | | |
|  | | Antimalarial for P. vivax | □ | **□** |  | | |
| **Remarks:** | |  |  |  |  | | |
|  | |  | Y | **N** |  | Y | **N** |
| **Recording and reporting** | | Real time (within 24 hours) | □ | □ | Fulfilment of the referral form | □ | □ |
|  | | Logistic recording | □ | □ | Drug register | □ | □ |
| **Supervision and monitoring** | | Fulfilled monitoring checklist | □ | □ |  |  |  |

**Remarks:**

# Secondary Data Guideline

1. **Monthly number of migrant population tested per site**

| 2020 | Jan | Feb | Mar | April | May | June | July | Aug | Sep | Oct | Nov | Dec |
| --- | --- | --- | --- | --- | --- | --- | --- | --- | --- | --- | --- | --- |
| Gaddaachauki |  |  |  |  |  |  |  |  |  |  |  |  |
| Jamunaha |  |  |  |  |  |  |  |  |  |  |  |  |
| 2021 | Jan | Feb | Mar | April | May | June | July | Aug | Sep | Oct | Nov | Dec |
| Gaddaachauki |  |  |  |  |  |  |  |  |  |  |  |  |
| Jamunaha |  |  |  |  |  |  |  |  |  |  |  |  |
| 2022 | Jan | Feb | Mar | April | May | June | July | Aug | Sep |  | | |
| Gaddaachauki |  |  |  |  |  |  |  |  |  |  |  |  |
| Jamunaha |  |  |  |  |  |  |  |  |  |  |  |  |

1. **Number of persons positive to screening that was diagnosed with malaria (P. vivax and P. falciparum)**

| 2020 | Jan | Feb | Mar | April | May | June | July | Aug | Sep | Oct | Nov | Dec |
| --- | --- | --- | --- | --- | --- | --- | --- | --- | --- | --- | --- | --- |
| Gaddaachauki |  |  |  |  |  |  |  |  |  |  |  |  |
| Jamunaha |  |  |  |  |  |  |  |  |  |  |  |  |
| 2021 | Jan | Feb | Mar | April | May | June | July | Aug | Sep | Oct | Nov | Dec |
| Gaddaachauki |  |  |  |  |  |  |  |  |  |  |  |  |
| Jamunaha |  |  |  |  |  |  |  |  |  |  |  |  |
| 2022 | Jan | Feb | Mar | April | May | June | July | Aug | Sep |  | | |
| Gaddaachauki |  |  |  |  |  |  |  |  |  |  |  |  |
| Jamunaha |  |  |  |  |  |  |  |  |  |  |  |  |

**CONSENT FORM**

After getting an explanation of the purpose, objective and benefit of the research, I am willing to participate in this study for providing information needed by Ms. Aney Rijal, a post graduate student studying International Master of Public Health at Universitas Gadjah Mada. I am agreeing to take part in this study. I will provide clear and true information based on my knowledge and experience. After signing this form, I consent to participate for interview in this study.

Name:

Age:

Gender:

Ethnicity:

Marital Status:

Highest level of Education:

Occupation/Designation:

Signature:

Date:

Signature of the witness:

Name of the witness:

# Key Informant Interview Guideline for the health worker in Health desk

**Exploring Barriers and Facilitators of Implementation Fidelity of the Malaria Screening Intervention in Point-of-entry Health Desk in Nepal-India Border**

Name of interviewee:

Type of community health worker:

Position:

Office:

Address:

| 1 | What is your role in this health desk? In which part of India does people often come from? How is the malaria screening done? (Probe: about the experience, from the standard operating procedure, frequency and coverage) |
| --- | --- |
| 2 | How do you perceive the strategies for screening in border screening? (Probe: Any need for further improvement in strategies) |
| 3 | Are the supplies (like RDT, PPE, Body temperature thermometer, registers) are available adequately? |
| 4 | Describe the communication process and reporting processes ? |
| 5 | What are the factors influencing the performance of HWs in malaria screening (health system and migrant population factors) |
| 6 | How political and financial support do you get for operating screening in point of entry? |
| 7 | How flexibly are you providing services amidst the burden of covid-19 and other diseases? |
| 8 | What are the challenges and problems in the standard operating protocol that is difficult to apply in reality? (Limitation, failures, mishaps or any bad practices reported) and enabling factors (probe: support, supervision, training, supplies, financing) |
| 9 | Is there any awareness-raising activity for migrant population so that they know about the screening services? |
| 10 | How is the coordination/collaboration between health desk and other stakeholders? |
| 11 | What motivates you to continue working as HW in border screening of malaria ? what do you recommend to continue working well. |

Name of Interviewer: Date of interview:

# Key Informant Interview Guideline for Municipality health coordinators

**Exploring Barriers and Facilitators of Implementation Fidelity of the Malaria Screening Intervention in Point-of-entry Health Desk in Nepal-India Border**

Name of interviewee:

Position:

Office:

Address:

| 1 | What is your role in this health desk? What do you think are the factors influencing the performance of HWs in malaria border screening? (Probe: from the standard operating procedure, workload, frequency and coverage) (from health system and migrant factors) |
| --- | --- |
| 2 | How is the malaria screening implemented and coordinated between health desk and municipality? (probe; communication process) |
| 3 | Explain the characteristics HWs you recruit for malaria intervention. |
| 4 | Do you have clearly defined tasks and roles for awareness raising activities ? |
| 5 | How you perceive the strategies for screening in border screening? (Probe: Any need for further improvement in strategies) |
| 6 | Describe how the supply system is managed to ensure consistency in their work |
| 7 | What is the burden of the malaria cases in this locality and how is the political and financial context in operating screening in point of entry? |
| 8 | To what extent was the process of inclusion, training, kit delivery, and follow-up visits implemented as planned? |
| 9 | What are the challenges (limitation, failures, mishaps or any bad practices reported) and enabling factors ? |
| 10 | How do you supervise the HW and how do you evaluate the HWs? |

Name of Interviewer: Date of interview:

:

# Key Informant Interview Guideline for district/provincial health directorate

**Exploring Barriers and Facilitators of Implementation Fidelity of the Malaria Screening Intervention in Point-of-entry Health Desk in Nepal-India Border**

Name of interviewee:

Position:

Office:

Address:

| 1 | What is your role in this health desk? What do you think are the factors influencing the performance of HWs in malaria border screening? (Probe: from the standard operating procedure, workload, frequency and coverage) (from health system and migrant factors) |
| --- | --- |
| 2 | How is the malaria screening implemented and coordination with health desk ? (probe; communication process) |
| 3 | Explain the characteristics HWs you recruit for malaria intervention. |
| 4 | Do you have clearly defined tasks and roles for awareness raising activities ? |
| 5 | How you perceive the strategies for screening in border screening? (Probe: Any need for further improvement in strategies) |
| 6 | Describe how the supply system is managed to ensure consistency in their work |
| 7 | What is the burden of the malaria cases in this province/district and how is the political and financial context in operating screening in point of entry? |
| 8 | Is the malaria screening in point of entry is well adapted with other disease screening agenda? |
| 9 | To what extent was the process of inclusion, training, kit delivery, and follow-up visits implemented as planned? |
| 10 | What are the challenges (limitation, failures, mishaps or any bad practices reported) and enabling factors ? |
| 11 | How do you supervise the HW and how do you evaluate the HWs? |

Name of Interviewer: Date of interview:

# Key Informant Interview Guideline for security officials

**Exploring Barriers and Facilitators of Implementation Fidelity of the Malaria Screening Intervention in Point-of-entry Health Desk in Nepal-India Border**

Name of interviewee:

Position:

Office:

Address:

| 1 | What is your role in this health desk? What do you think are the factors influencing the performance of HWs in malaria border screening? (Probe: from the standard operating procedure, workload, frequency and coverage) (from health system and migrant factors) |
| --- | --- |
| 2 | How is the malaria screening implemented and coordination with health desk ? (probe; communication process) |
| 3 | How do you perceive the strategies for screening in border screening? (Probe: Any need for further improvement in strategies) |
| 4 | What is the burden of the imported malaria case? and how is the political commitment and financial situation in operating screening in point of entry? |
| 5 | Is the malaria screening in point of entry is well adapted with other disease screening agenda? |
| 6 | What are the challenges (limitation, failures, mishaps or any bad practices reported) and enabling factors |
| 7 | Is there any awareness-raising activity for migrant population so that they know about the screening services? |
| 8 | What are the challenges in the standard operating protocol that is difficult to apply in reality? |

Name of Interviewer: Date of interview:

# Key Informant Interview Guideline for stakeholder of Epidemiology and Disease Control Division

**Exploring Barriers and Facilitators of Implementation Fidelity of the Malaria Screening Intervention in Point-of-entry Health Desk in Nepal-India Border**

Name of interviewee:

Position:

Office:

Address:

| 1 | What is your role in this health desk? What do you think are the factors influencing the performance of HWs in malaria border screening? (Probe: from the standard operating procedure, workload, frequency and coverage) |
| --- | --- |
| 2 | How is the malaria screening implemented and coordination with health desk ? (probe; communication process) |
| 3 | How long is Nepal going to continue the point of entry malaria screening? |
| 4 | How you perceive the strategies for screening in border screening? (Probe: Any need for further improvement in strategies) |
| 5 | What is the burden of the imported malaria case? and how is the political commitment and financial situation in operating screening in point of entry? |
| 6 | Is the malaria screening in point of entry is well adapted with other disease screening agenda? |
| 7 | To what extent was the process of inclusion, training, kit delivery, and follow-up visits implemented as planned? |
| 8 | What is the plan of EDCD to accelerate the malaria screening in border area? |
| 9 | Compared to past, what do feel about the current health desk providing integrated health services? |
| 10 | What are the challenges (limitation, failures, mishaps or any bad practices reported) and enabling factors |
| 11 | Is there any awareness-raising activity for migrant population so that they know about the screening services? |
| 12 | How is the coordination/collaboration between health desk and EDCD? |

Name of Interviewer: Date of interview:

# In-depth Interview Guideline for Migrant population

**Exploring Barriers and Facilitators of Implementation Fidelity of the Malaria Screening Intervention in Point-of-entry Health Desk in Nepal-India Border**

Name of interviewee:

Age:

Gender:

Address in Nepal: Municipality: Ward No: District:

Point of Entry Border: District:

Address in India:

| 1 | In which part of India did you come from? Where do you work and how is the environment to live at that part of India? Is it clean and is there any inducing tropical environment that people suffer from various infectious and specifically vector borne disease? |
| --- | --- |
| 2 | Screening carried out on routine basis/ on an ad hoc/ after a public health event has occurred? |
| 3 | How you perceive the strategies for screening in border screening? (Probe: ask if there’s any burden) |
| 4 | How comfortable do you feel while seeking health services? if not what is the reason? |
| 5 | How is the attitude of HWs and volunteers in the point of entry health desk? |
| 6 | Compared to past, what do feel about the current health desk providing integrated health services? |
| 7 | What challenges do you face while screening? |
| 8 | How do you get information about screening in border area? probe: knowledge about vector borne diseases |
| 9 | What do think can be done to reduce malaria burden among migrant population? |

Name of Interviewer:

Date of interview:
