# Supplementary material for: Exploring barriers and facilitators in implementation fidelity of malaria screening intervention at Nepal-India border point-of-entry health desks-A mixed method study
Source: PLoS One. 2025 May 7;20(5):e0323116. doi: 10.1371/journal.pone.0323116 (PMC12057938; doi:10.1371/journal.pone.0323116)
Supplement: Attachment — Submitted filename: Response to the reviewers_edited.docx [file pone.0323116.s003.docx]

**Plos One**

**COVER LETTER**

Date: 29^th^ July 2024

To,

The Editor in Chief,

PLOS One

Dear Sir/Madam,

Firstly, thank you for the reviews and suggestions. I am submitting the revised manuscript entitled “Exploring Barriers and Facilitators in Implementation Fidelity of Malaria Screening Intervention at Nepal-India Border Point-of-entry Health Desks”.

I declare that the present work is original, and no part of the manuscript has been published before, nor is any part of it under consideration for publication in another journal.

I have added the response to the reviewer attached below. We don’t have any disclosure to make. If you feel that the manuscript requires further revision, please let me know.

We are looking forward to hearing from you.

Yours Sincerely,

Name of the corresponding author: Aney Rijal

ORCID id: [0000-0003-0653-4823](https://orcid.org/0000-0003-0653-4823?lang=en)

Affiliation: Universitas Gadjah Mada, Yogyakarta, Indonesia,

HERD International, Lalitpur, Nepal

Email: rijal.aney@gmail.com

Phone: +9779849055766

**Response to the reviewer**

1. Please ensure that your manuscript meets PLOS ONE's style requirements, including those for file naming. The PLOS ONE style templates can be found at 
<https://journals.plos.org/plosone/s/file?id=wjVg/PLOSOne_formatting_sample_main_body.pdf> and 
<https://journals.plos.org/plosone/s/file?id=ba62/PLOSOne_formatting_sample_title_authors_affiliations.pdf>

Response: Thank you. We ensure that the manuscript meets PLOSOne’ style.

2. Please update your submission to use the PLOS LaTeX template. The template and more information on our requirements for LaTeX submissions can be found at <http://journals.plos.org/plosone/s/latex>.

**Response:** Okay, thank you. We have upload the tex file.

3. We note that the grant information you provided in the ‘Funding Information’ and ‘Financial Disclosure’ sections do not match.

When you resubmit, please ensure that you provide the correct grant numbers for the awards you received for your study in the ‘Funding Information’ section.

**Response:** I would like to clarify that the funding provided is not in the form of a grant for research purposes, but rather as a postgraduate scholarship. There is no specific funding has been allocated for research activities.

4. When completing the data availability statement of the submission form, you indicated that you will make your data available on acceptance. We strongly recommend all authors decide on a data sharing plan before acceptance, as the process can be lengthy and hold up publication timelines. Please note that, though access restrictions are acceptable now, your entire data will need to be made freely accessible if your manuscript is accepted for publication. This policy applies to all data except where public deposition would breach compliance with the protocol approved by your research ethics board. If you are unable to adhere to our open data policy, please kindly revise your statement to explain your reasoning and we will seek the editor's input on an exemption. Please be assured that, once you have provided your new statement, the assessment of your exemption will not hold up the peer review process.

**Response:** Minimal data sets supporting the conclusions of this article are included within the article and its supplementary files. This includes the values used to build graph and the values used to share about the findings.

We have also uploaded the supporting information files which are compressed in zip.

5. We note that several of your files are duplicated on your submission. Please remove any unnecessary or old files from your revision, and make sure that only those relevant to the current version of the manuscript are included.

**Response:** Thank you, we have done the needful by removing the duplicated submission files.

**Reviewer #1**: I would like to acknowledge all the authors of this paper for their hard work on this important issue. Saying that, I have few comments.
1. In the title, it is better to include the design of the study (mixed method)
**Response:** Thank you, We have revised as suggested.

2. Research gaps are not adequately addressed in the introduction part of the study.
**Response:** Thank you. We revised the introduction, The introduction includes the elaborated rationale now.

3. In methods
- The study setting, it is better to say "the study is carried out" instead of "we carried out."

**Response:** Thank you. The phrase is edited.

4. Data collection and analysis, How do you maintain the quality of the data since you are using secondary data? For the qualitative part of your study, the researchers used key-informant interviews and FGDs, but the number of key-informant interviews and FGDs conducted in the study is not indicated. At last, I am not interested in the way quantitative data are analyzed.

**Response:** Thank you. The secondary data was taken from the Epidemiology and Disease Control Division, Ministry of Health, Nepal. The data is real-time data and verified based on the case-based investigation. In terms of malaria testing data, it was obtained reviewing the malaria register. The malaria register provides information about the cases identified at POE, which was cross-verified based on the line listing data provided by EDCD.

4. The result part of the study need more work.

**Response:** We have tried to present the finding in concise, while few themes were rearranged for better understanding. We also rewrote the descriptions for some findings as per the feedback provided.

Reviewer #2: COMMENTS
1: It would be helpful to include a sketch map showing the locations
**Response**: Thank you. We inserted a sketch map on the study setting of Methodology in page no. 7.

2: The recommendation should be more specific, wherever possible it should be define to which authority they are targeted.
**Response**: Thank you. We revised the recommendations to be more specific. Please see page no. 29, last paragraph of the discussion.

3: The abbreviation for point of entry (POE)should be defined in the Abstract and at first mention in the text and should be consistent ( POE or PoE)
**Response:** We revised as suggested.

4: Regarding concerns about RDTs. This is an important issue that should be further clarified: is there any technical confirmation of the true reliability of the tests? What measures are recommended to address this problem.

**Response:** It is possible that the antibody test was procured or used at Point of Entry. Therefore, it is necessary to ensure the availability of antigen test kits, and positive antigen tests should be confirmed with microscopic testing.

Minor edits:
64: risk=the risk
67: an= a standard
68: the points of entry
167: screening was not done
174: when a person
399: Study=The study

**Response:** Thank you. We edited the writing mistakes as suggested.

Reviewer #3: Dear Authors,
Your manuscript has a strong foundation, but there are some areas that could benefit from further refinement. Here are some specific comments:

**Response:** Thank you for your appreciation.

1- Consider revising the language throughout the manuscript to ensure clear and impactful communication of your findings.
**Response:** Thank you. We have re-done English editing in this revised manuscript.

2- The abstract could be improved to more effectively convey the purpose and key takeaways of your research.
**Response:** Thank you. We revised the abstract to have more effective purpose statement and key takeaway messages from the research.

3- Some of the data, particularly regarding incidence and prevalence, may require updating with more recent information.
**Response:** Thank you, We have replaced with the updated incidence of malaria in introduction section.

4- The positive number of cases is related to increased transmission or increase/improvement of testing?

**Response:** Thank you for your question. Nepal is currently in the elimination phase of malaria. The strategies for testing have been intensified at health facility levels, points of entry (POE), and within communities through active case-based testing. Consequently, we can assert that the testing capabilities have improved. We clarify this by adding the sentence on page 4, line 92-95.

5- Please ensure consistency in the fiscal year (FY) used when presenting data on P. falciparum infection and total malaria cases. When you talk about P.falciparum infection the baseline is 2019/20-2020/21 FY year, but when talk about the total number of malaria cases your FY is 2020/21-2021/22. please unify the information to have a better interpretation.
**Response:** Thank you for your valuable comment. We have edited and added the data. Please see line 98-100.

6- Please clarify how the data presented in lines 61-63 connects to the subsequent sections of the manuscript.
**Response:** We have further elaborated on cases imported from India and other parts of world. Nepal shares a border with India, and the imported cases have been found not to originate from adjacent states of India, but rather from states such as: Maharashtra and Gujarat, which are of particular interest as border malaria burden further into achieving elimination. We clarify this on the sentence on page 4, line 80-81, line 101-103 and line 109-111.

7- Is the novelty of your study specific to Nepal, or does it hold broader implications for other border regions?
**Response:** The novelty of the study specific to Nepal has been further elaborated the introduction section. Please refer to page 6, line 141-145.

8- Please introduce any abbreviations used in the text upon first mention (e.g., EDCD, IEC, CDO).

**Response**: Thank you. We revised it as suggestion.
9- Please specify the method used to confirm positive malaria cases.

**Response:** The positive malaria cases are confirmed through RDT and microscopic tests when the patients come under testing at POE and in health facilities and active case detected at community level, which is also mentioned in page 4, line 93-94.

Meanwhile, secondary data were collected for the purpose of this study. This aim is not to conduct screening itself, but rather to analyse secondary data while concurrently engaging in qualitative inquiries with multiple stakeholders.

10- The data presented in lines 136-138 could benefit from further explanation to enhance reader understanding.

**Response:** Those data represents the total number of malaria cases identified in the previous year in Sudurpaschim and Lumbini province, which couldn’t provide the proper idea about total percentage of imported malaria cases, hence, the finding had to be removed.

11- Clarify whether the "International Health Regulations 2005" are considered international or local regulations
**Response:** Both national and international regulations were considered and is elaborated in the discussion. Please see page 28, line 682 to 684.

12-Adding a section discussing the limitations of your study would further strengthen your work.
By addressing these points, you can significantly improve the overall clarity, strength, and impact of your manuscript.

**Response:** The limitation section has been added. Please see page 32, line 782-790.
