# Supplementary material for: Exploring barriers and facilitators in implementation fidelity of malaria screening intervention at Nepal-India border point-of-entry health desks-A mixed method study
Source: PLoS One. 2025 May 7;20(5):e0323116. doi: 10.1371/journal.pone.0323116 (PMC12057938; doi:10.1371/journal.pone.0323116)
Supplement: Attachment — Submitted filename: Response to the reviewers_01.11.2025.docx [file pone.0323116.s004.docx]

**Plos One**

**COVER LETTER**

Date: 30^th^ December 2024

To,

The Editor in Chief,

PLOS One

Dear Sir/Madam,

Firstly, thank you for the reviews and suggestions. We are submitting the revised manuscript entitled “Exploring Barriers and Facilitators in Implementation Fidelity of Malaria Screening Intervention at Nepal-India Border Point-of-entry Health Desks: A Mixed Method Study”.

We declare that the present work is original, and no part of the manuscript has been published before, nor is any part of it under consideration for publication in another journal.

This research was a part of postgraduate thesis. The scholarship for this degree was funded by the UNICEF/UNDP/World Bank/WHO Special Programme for Research and Training in Tropical Disease at the World Health Organization (TDR) and Universitas Gadjah Mada, Indonesia. The funding is not specifically allocated for research activities. Also, the funders had no role in study design, data collection and analysis, the decision to publish, or the preparation of the manuscript. The funder provided support in the form of salaries for authors but did not have any additional role.

The supporting files attached consists of minimal data set as well as the tool for collecting epidemiological records of positive malaria cases.

We have added the response to the reviewer attached below. We don’t have any disclosure to make. If you feel that the manuscript requires further revision, please let us know.

We are looking forward to hearing from you.

Yours Sincerely,

Name of the corresponding author: Aney Rijal

ORCID id: [0000-0003-0653-4823](https://orcid.org/0000-0003-0653-4823?lang=en)

Affiliation: Universitas Gadjah Mada, Yogyakarta, Indonesia,

HERD International, Lalitpur, Nepal

Email: rijal.aney@gmail.com

Phone: +9779849055766

**Response to the reviewer**
1. The use of the track changes feature has made the document overwhelming, as nearly all parts are highlighted in red, making it difficult to discern the updates. While I tried my best to interpret the changes, the process was not straightforward.
**Response:** We appreciate your effort. We have attached both track change and clean version of the manuscript. This could bring some clarity on reviewing the changes.

2. I did not receive a clear response regarding suggestion 3. Kindly provide feedback on this point in a more structured and transparent manner.
**Response:** There is not availability of malaria incidence data for year 2024. So, we are mentioning data of fiscal year 2022/23.

3. The phrase "any part of the world" is vague and requires clarification to ensure the statement is clear and meaningful.
**Response:** This has been revised already.

4. Please follow standard formatting conventions by italicizing all species names used in the manuscript.
**Response:** Thank you. We have revised as per the standard format.

5. Include details about the suppliers of the kits used for detection in the methods section.
**Response:** Thank you. This sentence has been added in study setting, page no. 3, in line 111-112.

“These kits are procured through Management Division in coordination with Epidemiology and Disease Control Division (EDCD)”

6. While the introduction has been updated, the references associated with this section have not been revised accordingly.

**Response:** Thank you for pointing out. We have updated the references now.

7. Please ensure consistency between the text and the references regarding the updated data for incidence and prevalence.

**Response:** Thank you. We have cross-checked the references and revised.

8. In your response, you mentioned the use of "secondary data." Could you elaborate on the meaning of this term and provide further context?

**Response:** Thank you. We have clarified about secondary data in manuscript. In page no. 3, in line 152-152, under data collection and analysis.

“Secondary data on total number of tests conducted per month and cases of malaria identified at border points were taken at two phases.”
